# Supplementary material for: A protein-proximity screen reveals Ebola virus co-opts the mRNA decapping complex through the scaffold protein EDC4
Source: Nat Commun. 2025 Sep 26;16:8485. doi: 10.1038/s41467-025-63392-4 (PMC12475165; doi:10.1038/s41467-025-63392-4)
Supplement: Supplementary file 1 — Supplementary Information [file 41467_2025_63392_MOESM1_ESM.pdf]

A protein-proximity screen reveals Ebola virus co-opts the mRNA decapping complex through the scaffold protein EDC4

Callie J. Donahue, Aditi Kesari, Naveen Thakur, Ling Wang, Sarah Hulsey Stubbs, Caroline G. Williams, Sandhya Bharti Sharma, Cara D. Kirby, Daisy W. Leung, Uma K. Aryal, Christopher F. Basler, Douglas J. LaCount and Robert A. Davey

Supplementary Information.

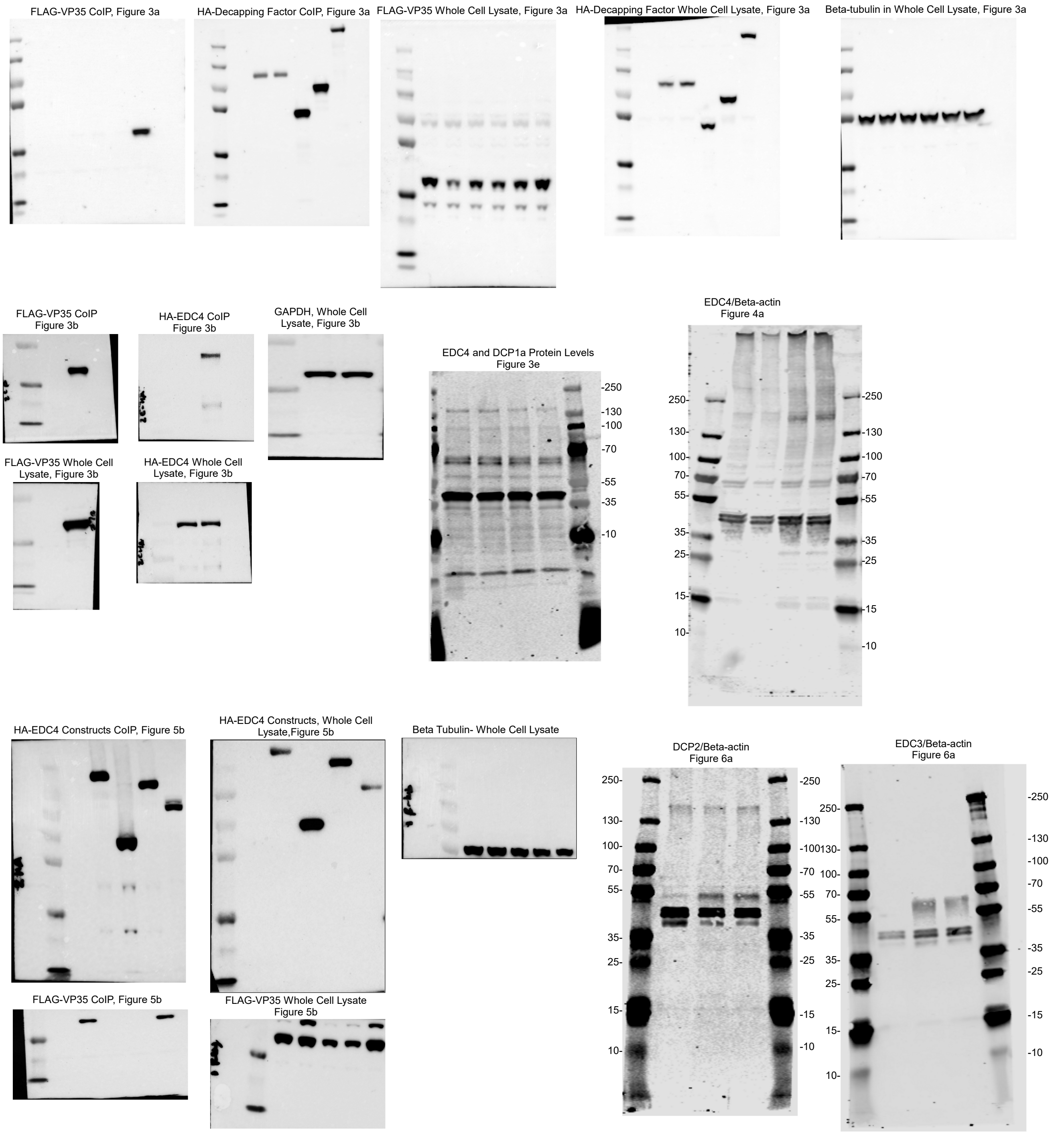

Supplementary Figure 1: Images of complete blots for coimmunoprecipitation and siRNA depletion experiments. Separate immunoblots were performed for co-immunoprecipitation experiments and probed as indicated. Blots correspond to the indicated figures.

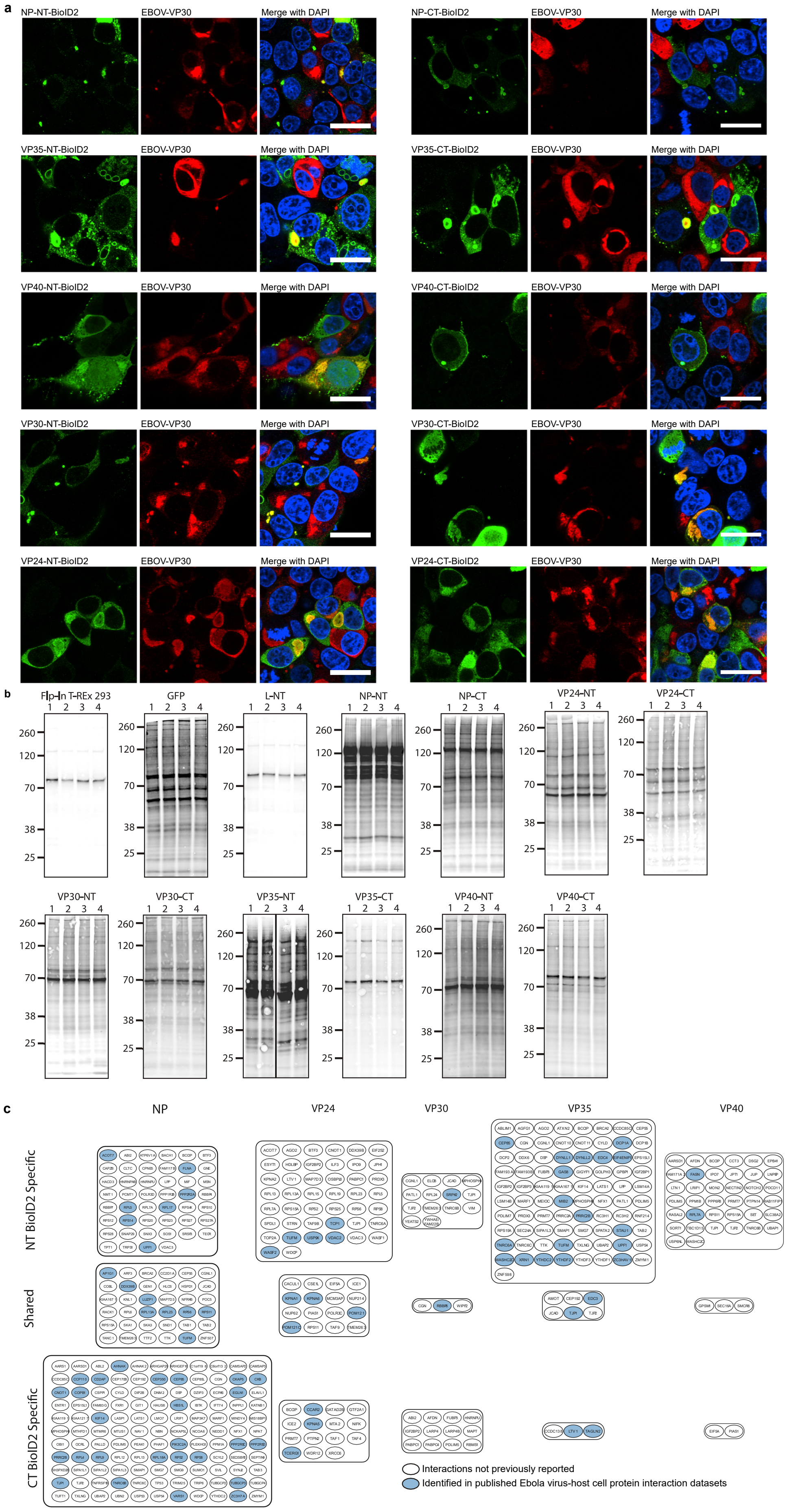

Supplementary figure 2: a Cellular localization of BioID2-tagged constructs. HEK293T cells were transfected with N and C-terminal BioID2 tagged expression constructs and challenged with EBOV at an MOI of 1, then fixed at 20 hpi and stained for BioID2 protein (green, first panel set) and EBOV VP30 (red, second panel set). Imaging was by a confocal microscope. Each channel is combined together with Hoechst 33342 staining of cell nuclei (blue) in the third set of panels. Scale bar = 25 µm. **b** Abundance of biotinylated proteins in cells expressing viral BioID2 constructs. Each BioID2 screen was performed in quadruplicate. The top left shows endogenous biotinylation in the Flp-In TREx 293 cell line, which expresses no BioID2 construct, and the following panel represents non-specific biotinylation by the GFP-BioID2 construct. Subsequent panels demonstrate biotinylation of the indicated constructs. Following processing, samples were transferred to nitrocellulose membranes and stained with fluorescently labeled streptavidin. **c** Interactions identified specifically by viral proteins fused to BioID2 at their N- or C-termini (NT and CT, respectively) or by both constructs (Shared, center row). Ovals shaded blue indicate proteins identified in other published studies of EBOV-host interactions.

a

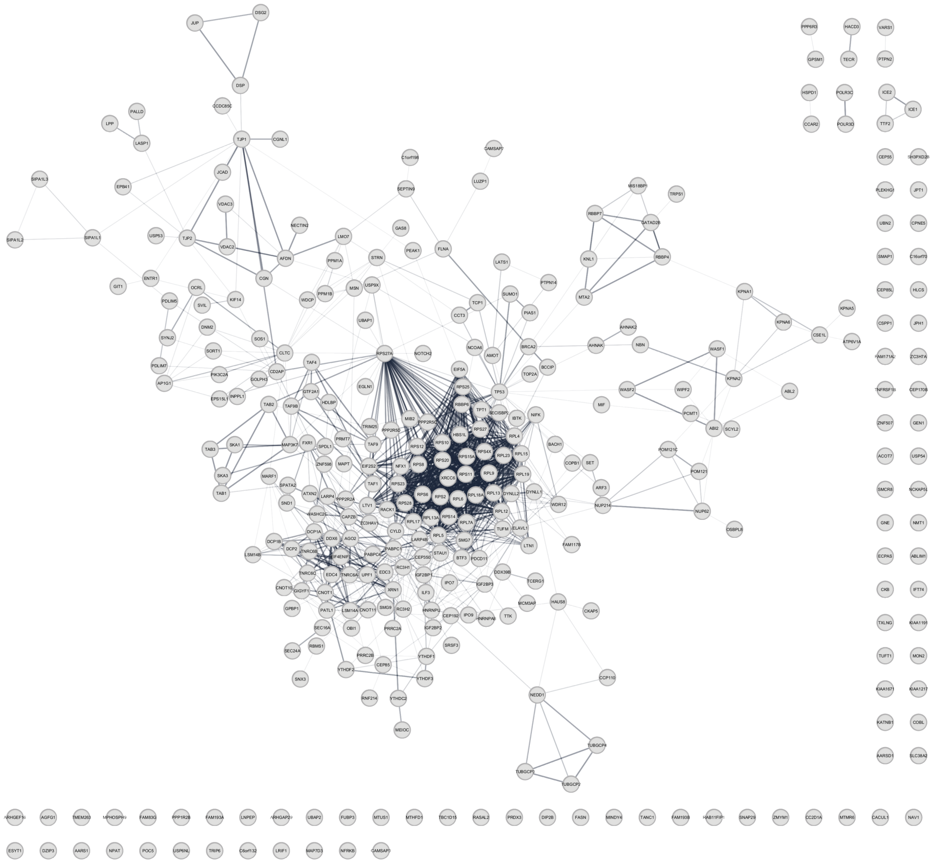

b

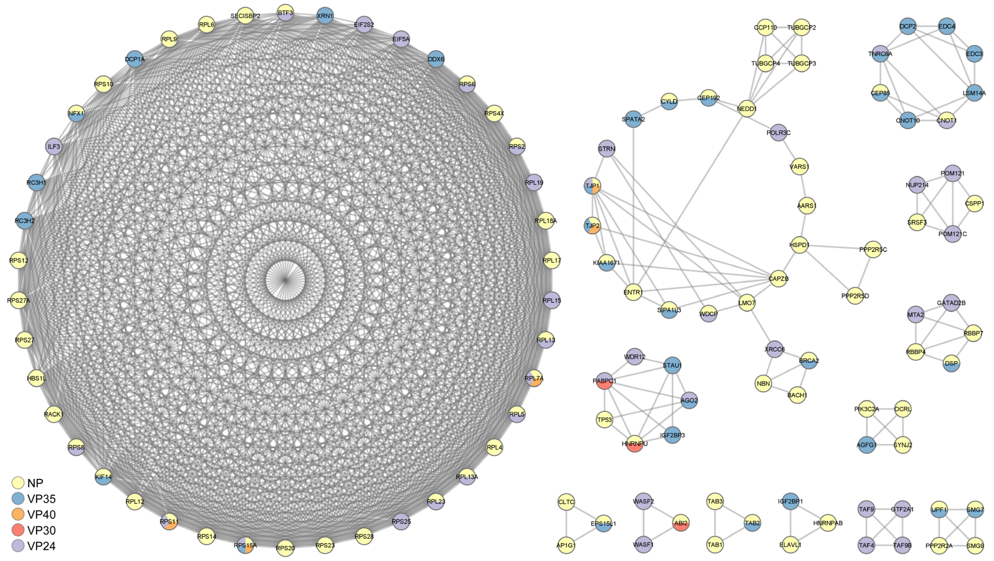

Supplementary Figure 3. a STRING network analysis of BioID hits. STRING v12.0 was used to map BioID hits to the STRING database. The thickness of the black edge lines correlates to edge confidence scoring (0.4-1.0) while grey circles represent protein hits from the screen. Grey circles **(**right side and bottom) not connected by dark grey lines had no interactions in the STRING database linking them to other proteins in the network. **b** MCODE clustering of hits from viral protein BioID2 screen. Hits from BioID2 screens were subjected to Metascape analysis which relies on MCODE analysis to identify subnetworks. MCODE is an edge-density based algorithm that prioritizes complex identification based on the number of connections between input proteins. Node colors indicate the viral protein that identified each hit. The large, interconnected network at left is composed of ribosomal proteins.

a

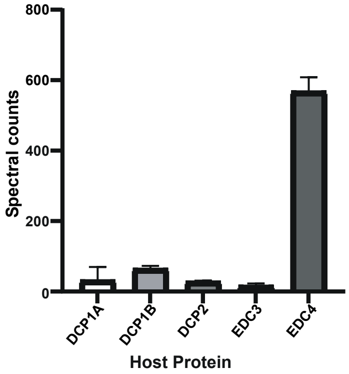

b

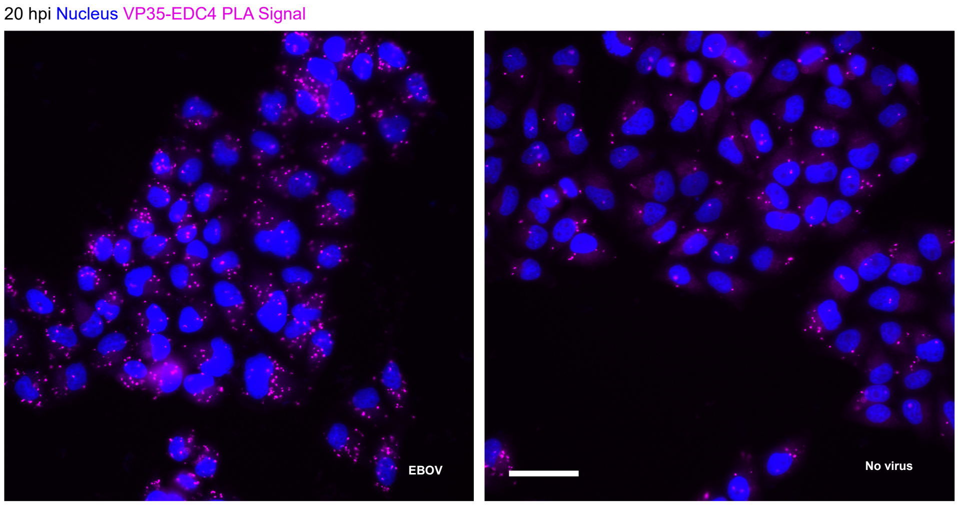

Supplementary figure 4: a Average spectral counts from four replicates for each decapping protein are shown in the graph. EDC4 was the most abundantly labeled protein of the decapping complex proteins identified. **b** Expanded view of PLA of EDC4 and VP35 in EBOV-infected cells (left panel) and mock infected (right panel). Single plane image taken at 20X. Scale bar = 25μm.

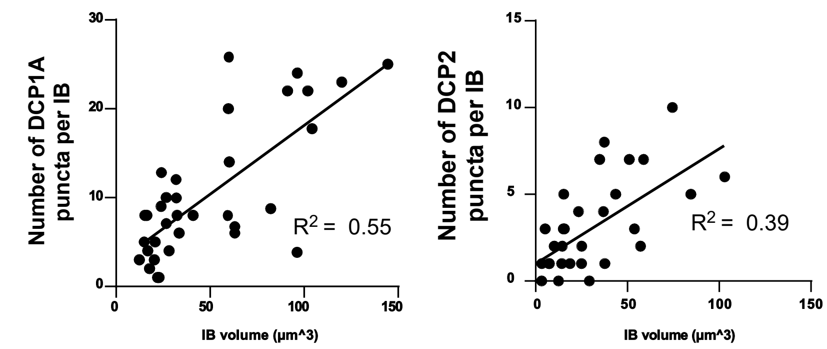

Supplementary figure 5: Quantification of decapping factor localization to inclusion bodies (IB). Super-resolution confocal microscopy Z-stack images of EBOV-challenged cells were stained for VP35 and **a** DCP1A or **b** DCP2 and puncta colocalizing in VP35 stained inclusion bodies measured in Imaris. At least 20 inclusion body (IB) volumes of varying sizes were measured per sample, and the number of each protein puncta per inclusion body were calculated. Simple linear regression was performed yielding R^2 values of 0.83 and 0.39 for DCP1A and DCP2 correlation with VP35 respectively.
