# Supplementary material for: A protein-proximity screen reveals Ebola virus co-opts the mRNA decapping complex through the scaffold protein EDC4
Source: Nat Commun. 2025 Sep 26;16:8485. doi: 10.1038/s41467-025-63392-4 (PMC12475165; doi:10.1038/s41467-025-63392-4)
Supplement: Supplementary file 2 — Description Of Additional Supplementary File [file 41467_2025_63392_MOESM2_ESM.pdf]

Description of additional supplementary files

Supplementary Data 1

Title: High-confidence Host Protein Interactions Identified by EBOV BioID2 Screens
Description: This file contains the final set of 441 host proteins identified through proximity-dependent biotinylation using BioID2-tagged EBOV proteins. The dataset includes hits derived from two independent SAINTExpress analyses using GFP-BioID2 and other EBOV proteins as background controls. Both N- and C-terminally tagged constructs were analyzed to capture interactions dependent on protein orientation. Confidence scores and replicate data are included to support prioritization of interactors.

Supplementary Data 2

Title: EBOV BioID2 Hits Overlapping with Functional Genetic Screens
Description: This table lists BioID2 hits that overlap with previously published CRISPR and siRNA screens for EBOV host factors. Seven proteins identified in our screen were previously implicated in EBOV replication by functional loss-of-function assays. This cross-validation highlights the biological relevance of our proximity labeling dataset. References and screen-specific annotations are included.

Supplementary Data 3

Title: EBOV BioID2 Hits Found in EBOV Virions
Description: This dataset provides host proteins identified in the BioID2 screen that were also found in purified EBOV virions via mass spectrometry. Five overlapping proteins are reported, suggesting physical incorporation or strong association with virus particles. These data support a role for these host factors during late stages of viral assembly or budding. Spectral count comparisons and viral association notes are included.

Supplementary Data 4

Title: Cross-reference of EBOV BioID2 Hits with Published Proteomics Studies
Description: This file summarizes the overlap between the BioID2 dataset and several large-

scale EBOV proteomics studies. A total of 81 interactions were found to be previously reported, providing strong external validation. References include AP-MS, split-TurboID, and VP40 BioID datasets. Each entry includes source study identifiers and hit annotations.

Supplementary Data 5

Title: SAINT Scores Used as Prizes in PCSF Network Analysis
Description: This file includes the SAINTExpress-derived interaction scores used as "prize values" for the Prize Collecting Steiner Forest (PCSF) algorithm. Scores were assigned to each protein to guide optimal subnetwork generation within the HIPPIE protein-protein interaction network. Duplicate hits from N- and C-terminal constructs were collapsed using the highest confidence score. These data informed the network shown in Figure 2.

Supplementary Data 6

Title: Steiner Forest Subnetworks Connecting EBOV BioID2 Hits
Description: This table includes all host proteins and interaction edges included in the optimal Steiner forest subnetwork derived using PCSF. Each interaction includes confidence metrics, edge weights, and connectivity information. A total of 335 proteins are connected within this high-confidence network. These subnetworks were used to construct the functional clustering and viral protein annotation presented in Figure 2d–e.

Supplementary Data 7

Title: Viral Protein Annotations for Host Interactors in PCSF Network
Description: This file maps each host protein node in the PCSF-derived network to its interacting EBOV protein(s). Annotation scores reflect normalized SAINT values and are scaled for each viral protein interaction. Multivalent host interactions (with more than one viral protein) are indicated. This dataset was used to color annotate Figure 2d to identify virus proteins interacting with the networked host proteins.

Supplementary Data 8

Title: Functional Enrichment of PCSF Network Clusters

Description: Functional enrichment analysis was performed on each PCSF-derived cluster using GO, Reactome, and KEGG databases. This file includes enrichment terms, adjusted p-values, and associated cluster IDs. P-values were calculated by EnrichR using Fisher’s exact test. These annotations provide insight into the biological processes and cellular components most affected by EBOV interactions. Clusters are aligned with those annotated in Figure 2e.

Supplementary Data 9

Title: RNA FISH Probe Sequences for Detection of EBOV Transcripts

Description: This dataset contains sequences of primary and secondary probes used in RNA FISH to detect EBOV NP and GP mRNAs. Primary probes contain an additional 20-nucleotide flap for secondary probe hybridization, which is conjugated to Cy5. Probes were used for quantifying viral RNA levels in infected cells after siRNA or truncation mutant treatments. Flap sequences and labeling chemistry details are included for replication.
