# Supplementary material for: A protein-proximity screen reveals Ebola virus co-opts the mRNA decapping complex through the scaffold protein EDC4
Source: Nat Commun. 2025 Sep 26;16:8485. doi: 10.1038/s41467-025-63392-4 (PMC12475165; doi:10.1038/s41467-025-63392-4)
Supplement: Supplementary file 12 — Reporting summary [file 41467_2025_63392_MOESM12_ESM.pdf]

nature portfolio

Corresponding author(s): Robert Davey and Douglass LaCount

Last updated by author(s): Aug 18, 2025

Reporting Summary

Nature Portfolio wishes to improve the reproducibility of the work that we publish. This form provides structure for consistency and transparency in reporting. For further information on Nature Portfolio policies, see our Editorial Policies and the Editorial Policy Checklist.

Statistics

For all statistical analyses, confirm that the following items are present in the figure legend, table legend, main text, or Methods section.

n/a	Confirmed	
☐	☒	The exact sample size (n) for each experimental group/condition, given as a discrete number and unit of measurement
☐	☒	A statement on whether measurements were taken from distinct samples or whether the same sample was measured repeatedly
☐	☒	The statistical test(s) used AND whether they are one- or two-sided<br>*Only common tests should be described solely by name; describe more complex techniques in the Methods section.*
☐	☒	A description of all covariates tested
☐	☒	A description of any assumptions or corrections, such as tests of normality and adjustment for multiple comparisons
☐	☒	A full description of the statistical parameters including central tendency (e.g. means) or other basic estimates (e.g. regression coefficient) AND variation (e.g. standard deviation) or associated estimates of uncertainty (e.g. confidence intervals)
☐	☒	For null hypothesis testing, the test statistic (e.g. F, t, r) with confidence intervals, effect sizes, degrees of freedom and P value noted<br>*Give P values as exact values whenever suitable.*
☐	☒	For Bayesian analysis, information on the choice of priors and Markov chain Monte Carlo settings
☒	☐	For hierarchical and complex designs, identification of the appropriate level for tests and full reporting of outcomes
☒	☐	Estimates of effect sizes (e.g. Cohen's d, Pearson's r), indicating how they were calculated

Our web collection on statistics for biologists contains articles on many of the points above.

Software and code

Policy information about availability of computer code

Data collection	R was used for data curation.
Data analysis	Analysis of data was performed using custom code written by the first author, using open-source packages on R. This is made publically available, including example datasets through https://github.com/cjdonahue14/BioID-Network-Analysis/tree/5 and as a DOI locked version on Zenodo https://doi.org/10.5281/zenodo.15708552

For manuscripts utilizing custom algorithms or software that are central to the research but not yet described in published literature, software must be made available to editors and reviewers. We strongly encourage code deposition in a community repository (e.g. GitHub). See the Nature Portfolio guidelines for submitting code & software for further information.

Data

Policy information about availability of data

All manuscripts must include a data availability statement. This statement should provide the following information, where applicable:

- Accession codes, unique identifiers, or web links for publicly available datasets
- A description of any restrictions on data availability
- For clinical datasets or third party data, please ensure that the statement adheres to our policy

The full mass spectrometry data is made available on MASSive with accession dataset ID MSV000093669. Source data images are available on Zenodo under DOI

10.5281/zenodo.13145450 and https://doi.org/10.5281/zenodo.16618585 and in the Source data supplementary file. The data is provided with no restrictions.

Research involving human participants, their data, or biological material

Policy information about studies with human participants or human data. See also policy information about sex, gender (identity/presentation), and sexual orientation and race, ethnicity and racism.

Reporting on sex and gender	N/a
Reporting on race, ethnicity, or other socially relevant groupings	*Please specify the socially constructed or socially relevant categorization variable(s) used in your manuscript and explain why they were used. Please note that such variables should not be used as proxies for other socially constructed/relevant variables (for example, race or ethnicity should not be used as a proxy for socioeconomic status).*<br>*Provide clear definitions of the relevant terms used, how they were provided (by the participants/respondents, the researchers, or third parties), and the method(s) used to classify people into the different categories (e.g. self-report, census or administrative data, social media data, etc.)*<br>*Please provide details about how you controlled for confounding variables in your analyses.*
Population characteristics	*Describe the covariate-relevant population characteristics of the human research participants (e.g. age, genotypic information, past and current diagnosis and treatment categories). If you filled out the behavioural & social sciences study design questions and have nothing to add here, write "See above."*
Recruitment	*Describe how participants were recruited. Outline any potential self-selection bias or other biases that may be present and how these are likely to impact results.*
Ethics oversight	*Identify the organization(s) that approved the study protocol.*

Note that full information on the approval of the study protocol must also be provided in the manuscript.

Field-specific reporting

Please select the one below that is the best fit for your research. If you are not sure, read the appropriate sections before making your selection.

☒ Life sciences ☐ Behavioural & social sciences ☐ Ecological, evolutionary & environmental sciences

For a reference copy of the document with all sections, see nature.com/documents/nr-reporting-summary-flat.pdf

Life sciences study design

All studies must disclose on these points even when the disclosure is negative.

Sample size	Unless stated otherwise in figure legends, all experiments were performed with at least 3 technical replicates and then repeated with at least 3 independent experiments. For experiments measuring changes in cell infection, at least 20000 cells are surveyed in each well of plates with infectivity starting at 10% for the untreated control. In previous work performed by our group using mass spectrometry based screening (Batra et al, 2018) , we determined that running samples with at least three replicates was sufficient for achieving statistical significance.<br>For experiments examining colocalization of decapping proteins and VP35, at least 150 cells were imaged , which was found in previous studies to be sufficient for achieving significance in image analysis (Sakurai et al, 2017).
Data exclusions	For the majority of work, being microscopy image based using automated capture systems, images that have mechanical artifacts such as not in focus or loss of cells due to technical issues are rejected, though this occurred with less than 1% of images. All other images are used for analysis. For RT-qPCR replicates, samples that failed to amplify in both the target gene and housekeeping gene and were demonstrated to have little to no RNA in Trizol-extracted samples were excluded.
Replication	Experiments were performed at least 3 times before being reported. An outcome was reported when 3 replicates similar in direction and magnitude were identified. Covariates were controlled as described below. Experiments were performed with multiple frozen and thawed vials of cells, as well as multiple lots of siRNA and transfection reagents, and results were only reported when samples produced consistent results across these conditions.
Randomization	Samples are captured and then read-out by a computer which has no information on the treatment being evaluated. This makes the readout agnostic of the technician setting up the experiment.<br>Covariates such as time post transfection and time post infection were controlled by always performing experiments within certain time windows (20-24 hours post transfection, 16-20 hours post infection). Incubation variables for experiments involving infection were controlled by keeping samples consistently at 37 degrees celsius with 5% carbon dioxide supplementation except for when experimental work was performed in the hood and/or samples were transferred from BSL2 to BSL4 facilities. To control for cell growth and cell population numbers, cells were consistently seeded at consistent live cell count numbers for each respective experiment and were only seeded for experiments if overall population viability was found to be at least 90% by Trypan Blue staining.
Blinding	Data collection is performed blinded as it is collected by a computer and the analysis is then performed by a set computer program that does not understand information on what is being tested. No data points were empirically scored, and thus other forms of blinding were not relevant to this study.

Reporting for specific materials, systems and methods

We require information from authors about some types of materials, experimental systems and methods used in many studies. Here, indicate whether each material, system or method listed is relevant to your study. If you are not sure if a list item applies to your research, read the appropriate section before selecting a response.

Materials & experimental systems

n/a	Involved in the study
☐	☒ Antibodies
☐	☒ Eukaryotic cell lines
☒	☐ Palaeontology and archaeology
☒	☐ Animals and other organisms
☒	☐ Clinical data
☒	☐ Dual use research of concern
☒	☐ Plants

Methods

n/a	Involved in the study
☒	☐ ChIP-seq
☒	☐ Flow cytometry
☒	☐ MRI-based neuroimaging

Antibodies

Antibodies used

Anti-DCP1A (clone D6VR1, cat# 15365, lot# 1) anti-EDC4 (cat# 2548S, lot# 2), and anti-HA (clone C29F4, cat# 3724S, lot# 11) antibodies were purchased from Cell Signaling Technology (Danvers MA). Anti-DCP2 antibody (cat# HPA057676, lot# R80862) was developed by Prestige Antibodies and purchased from Sigma-Aldrich (St.Louis MO). Anti-DDX6 antibody (cat# PA5-18478, lot# WI3373004) was developed by Thermofisher (Waltham MA). For immunoblotting, anti-EDC4 antibody (cat# 17737-1-AP) was purchased from Proteintech (Rosemont IL) and anti-EDC3 antibody (cat# A13763, lot# 0065350201) was purchased from ABClonal (Woburn MA). Anti-β-actin antibody (cat# MAB8929, clone # 937215, lot# CJQV032104) and HSP60 antibody (cat# AF1800, lot# KRO0921121) was purchased from R&D Systems (Minneapolis MN). Alexa-Fluor 488 goat anti-rabbit (lot#2500542) , Alexa-Fluor 546 goat anti-mouse (lot# 2026145) as well as Alexa-Fluor 488 chicken anti-goat (lot# 2328940), Alexa-Fluor 546 donkey anti-rabbit (lot# 2411581) and Alexa-Fluor 637 donkey anti-mouse (lot# 2555690) antibodies were purchased from Invitrogen (Thermofisher Scientific, Waltham MA). Chicken anti-BioID2 primary antibody was purchased from BioFront Technologies (BioBID-CP-100). IRDye 800CW donkey anti-chicken secondary antibody (LI-COR Biotechnology, 925-32218), IRDye® 800CW Goat anti-Rabbit IgG Secondary Antibody (LI-COR Biotechnology, 925-32211, lot# D30627-01), and IRDye® 680RD Goat anti-Mouse IgG Secondary Antibody (LI-COR Biotechnology, 926-68070, lot# D30613-05). Proximity Ligation Analysis antibody Donkey Anti-Mouse MINUS probe (cat# DUO82004-30RXN; lot# SLBZ2997) and Donkey Anti-Rabbit PLUS probe (cat# DUO82002-30RXN; lot# SLCB6741) were purchased as parts of the Duolink Kit from Millipore Sigma (Burlington MA)

Validation

The EBOV VP35 antibody was verified by examining signal in the presence and absence of infection and exogenous expression of VP35 proteins. Decapping complex antibodies were verified by siRNA KD, immunofluorescence staining pattern (DDX6 antibody to EDC4 p-bodies), or by the manufacturer.

Eukaryotic cell lines

Policy information about cell lines and Sex and Gender in Research

Cell line source(s)

HeLa cells (female), VeroE6 (female) cells and HEK293T (female) cells were purchased from ATCC® (Manassas, VA). Flp-In™ T-REx™ 293 Cells (cat# R78007) were purchased from Fisher Scientific (Hampton NH).

Authentication

Cell lined were authenticated by SNP analysis. VeroE6 cells relied on fresh samples from ATCC.

Mycoplasma contamination

All cell lines are verified free of mycoplasma by PCR analysis

Commonly misidentified lines
(See ICLAC register)

Name any commonly misidentified cell lines used in the study and provide a rationale for their use.

Plants

Seed stocks

Report on the source of all seed stocks or other plant material used. If applicable, state the seed stock centre and catalogue number. If plant specimens were collected from the field, describe the collection location, date and sampling procedures.

Novel plant genotypes

Describe the methods by which all novel plant genotypes were produced. This includes those generated by transgenic approaches, gene editing, chemical/radiation-based mutagenesis and hybridization. For transgenic lines, describe the transformation method, the number of independent lines analyzed and the generation upon which experiments were performed. For gene-edited lines, describe the editor used, the endogenous sequence targeted for editing, the targeting guide RNA sequence (if applicable) and how the editor was applied.

Authentication

Describe any authentication procedures for each seed stock used or novel genotype generated. Describe any experiments used to assess the effect of a mutation and, where applicable, how potential secondary effects (e.g. second site T-DNA insertions, mosiacism, off-target gene editing) were examined.
